# Nomogram for Predicting Portal Vein Thrombosis in Cirrhotic Patients: A Retrospective Cohort Study

**DOI:** 10.3390/jpm13010103

**Published:** 2023-01-01

**Authors:** Jingnuo Ding, Fazhi Zhao, Youhan Miao, Yunnuo Liu, Huiting Zhang, Weifeng Zhao

**Affiliations:** 1 Department of Infectious Diseases, The First Affiliated Hospital of Soochow University, No. 188 Shizi Street, Gusu District, Suzhou 215000, China; 2 Department of Stomach Surgery, Sichuan Cancer Hospital & Institute, Chengdu 610041, China; 3 Department of Infectious Diseases, The Third Affiliated Hospital of Nantong University, Nantong 226006, China

**Keywords:** portal vein thrombosis, cirrhosis, nomogram, predictive model, survival curve

## Abstract

Aim: Portal vein thrombosis (PVT) is a common complication in cirrhotic patients and will aggravate portal hypertension, thus leading to a series of severe complications. The aim of this study was to develop a nomogram based on a simple and effective model to predict PVT in cirrhotic patients. Methods: Clinical data of 656 cirrhotic patients with or without PVT in the First Affiliated Hospital of Soochow University and The Third Affiliated Hospital of Nantong University from January 2017 to March 2022 were retrospectively collected, and all patients were divided into training, internal and external validation cohorts. SPSS and R software were used to identify the independent risk factors and construct a predictive model. We evaluated the predictive value of the model by receiver operating characteristic (ROC) curve, calibration curve, and decision curve analyses. The feasibility of the model was further validated in the internal and external cohorts. All enrolled patients were followed up to construct the survival curves and calculate the incidence of complications. Results: The predictors of PVT included serum albumin, D-dimer, portal vein diameter, splenectomy, and esophageal and gastric varices. Based on the clinical and imaging findings, the final model served as a potential tool for predicting PVT in cirrhotic patients, with an AUC of 0.806 (0.766 in the internal validation cohort and 0.845 in the external validation cohort). The decision curve analysis revealed that the model had a high level of concordance between different medical centers. There was a significant difference between the PVT and non-PVT groups in survival analyses, with *p* values of 0.0477 and 0.0319 in the training and internal validation groups, respectively, along with *p* value of 0.0002 in the external validation group according to log-rank test; meanwhile, the median survival times of the PVT group were 54, 43, and 40 months, respectively. The incidence of recurrent esophageal and gastric variceal bleeding (EGVB) during the follow-up showed significant differences among the three cohorts (*p* = 0.009, 0.048, and 0.001 in the training, internal validation, and external validation cohorts, respectively). Conclusion: The nomogram based on our model provides a simple and convenient method for predicting PVT in cirrhotic patients. Cirrhotic patients with PVT had a shorter survival time and were prone to recurrent EGVB compared with those in the non-PVT group.

## 1. Introduction

Portal vein thrombosis (PVT) refers to the presence of a thrombus in the main lumen of the portal vein, which can extend to the intrahepatic or extrahepatic vein branches such as the splenic vein and superior mesenteric vein. PVT is a common complication in cirrhotic patients, especially in advanced cirrhosis. The morbidity of PVT varies in different studies. A prospective study showed that the cumulative incidence of PVT during the first year and third year was 3.7% and 7.6%, respectively, and it increased with the progression of cirrhosis [1].

Recent studies have focused on the reasons why cirrhotic patients would undergo PVT, with consideration given to the decreased coagulation factors and platelet counts. Patients with cirrhosis have an unbalanced coagulation system, which can vary to promote bleeding or thrombotic tendency in different disease stages [2]. At present, the main mechanism of PVT involves decreased portal vein flow, hypercoagulation status, and damage to the vessel wall [3]. Various etiologies cause hepatocyte injury, inflammation, and edema, thus leading to pseudolobule formation and collection in connective tissues. When liver sclerosis deteriorates, the portal vein blood flow slows, and its pressure is consequently increased. Continuous high pressure will damage the vascular endothelium and cause local vascular injury. In addition, due to deficiency in immune modulation, patients with cirrhosis always have alterations in their intestinal flora and experience inflammatory injury, which will aggravate the prethrombotic state. A combination of the above factors may contribute to portal vein system thrombosis.

According to a recent study, the risk factors for PVT in cirrhosis include cryptogenic and metabolic-associated fatty liver disease-related cirrhosis; Child-Pugh grade B and C; esophageal varices grade equal to or greater than grade II; portal vein blood flow < 15 cm/s; large portosystemic collateral circulation with high blood flow; local vascular damage; previous splanchnic vein thrombosis; sclerotherapy of esophageal varices and splenectomy; inflammation of the portal vein, abdominal cavity, and intestinal tract; and partial splenic embolization. Most of these risk factors are associated with portal hypertension [3,4,5]. However, most clinicians have only focused on the most frequent complications of cirrhosis, such as esophageal and gastric variceal bleeding (EGVB), ascites, and hepatic encephalopathy (HE); however, PVT development and its potential damage to these individuals during their disease progression have not been paid much attention to.

Patients with PVT present with the absence of specific clinical manifestations and sometimes experience only mild abdominal pain or even no symptoms at all. PVT is usually discovered incidentally by imaging during a physical examination. However, if the thrombus obstructs not only the main trunk and branches of the portal vein but also spreads to the splenic vein and superior mesenteric vein, it will lead to a series of related complications, such as massive gastrointestinal bleeding, cavernous transformation of portal vein, intestinal ischemia and necrosis, multiple organ failure, and even death [3]. In addition, the current imaging techniques only detect the existing PVT. Although many prospective, retrospective, and cross-sectional studies have identified some high-risk factors for the formation of PVT [5,6,7,8], a simple and effective measure to predict PVT formation in cirrhotic patients is still lacking.

Therefore, considering the occult nature and severity of the disease, we aimed to construct a predictive model for PVT and design a nomogram to help clinicians identify the high-risk population of PVT among cirrhotic patients to improve their prognosis.

## 2. Materials and Methods

### 2.1. Patients

**Figure 1:**
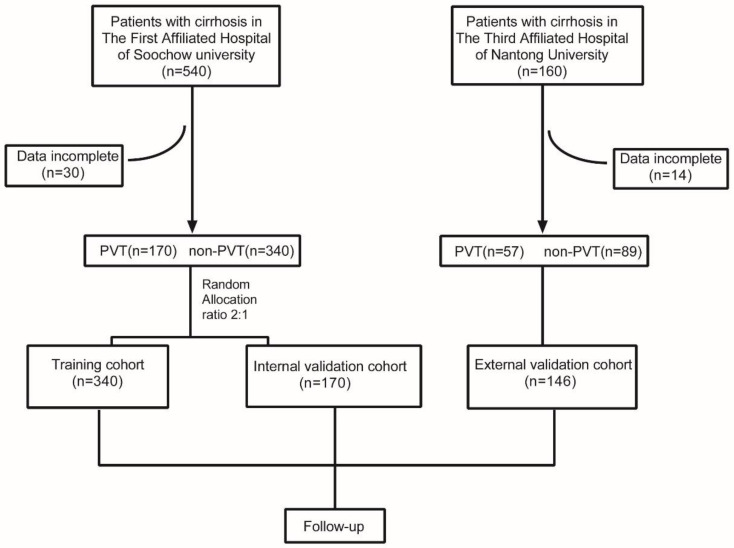
Flow diagram of the present study, PVT—portal vein thrombosis. Randomization was performed with R software.

Clinical data of 700 patients with cirrhosis treated in the Department of Infectious Diseases at the First Affiliated Hospital of Soochow University and The Third Affiliated Hospital of Nantong University from January 2017 to March 2022 were collected retrospectively. Forty-four patients were excluded from the study due to the missing data, and finally, there were 656 patients enrolled in the study. We used randomized assignment to divide the 510 patients from The First Affiliated Hospital of Soochow University into two groups: the training and internal validation cohorts (ratio 2:1). The training cohort contained 340 patients, and the internal validation cohort contained 170 patients. A total of 146 patients from The Third Affiliated Hospital of Nantong University were selected as the external validation cohort. The flow diagram is shown in Figure 1.

### 2.2. Inclusion and Exclusion Criteria

The inclusion and exclusion criteria were based on the consensus and guidelines for the diagnosis and treatment of chronic viral hepatitis, alcoholic liver disease, autoimmune hepatitis, cirrhosis, and portal vein thrombosis in cirrhotic patients [4]. The diagnosis of PVT is mainly based on ultrasound, computed tomography (CT), and magnetic resonance imaging (MRI), while esophageal gastric varices are interpreted according to gastroscopy, CT, and MRI. The inclusion criteria are described below (1) Patients were older than 18 years of age. (2) Patients were diagnosed with cirrhosis according to the liver histology, imaging material, and laboratory examinations related to the guidelines of diagnosis of cirrhosis [9], with the specific criteria being as follows: i: histology consistent with cirrhosis; ii: endoscopic evidence of esophageal and gastric varices (except non-cirrhotic portal hypertension); iii: ultrasound, liver stiffness measurement, CT, and other imaging examinations suggesting liver cirrhosis or portal hypertension characteristics, such as splenomegaly, portal vein diameter ≥ 13 mm; iv: in the absence of histological, endoscopic, or imaging data, any 2 abnormalities suggesting cirrhosis (i.e., any 2 of platelet (PLT)< 100 × 10^9^/L with no clear explanation, serum albumin (Alb) < 35 g/L, excluding malnutrition or kidney disease, international normalized ratio (INR) > 1.3 or prolonged prothrombin time (PT) (suspend thrombolytic or anticoagulant drugs for more than 7 days), and APRI (AST/PLT) score > 2). (3) Imaging examinations indicated thrombosis in the portal vein system, including the main trunk and branches, with or without the splenic vein and superior mesenteric vein, in the PVT group, and imaging examinations showed that blood flow was unobstructed in the portal vein system without filling defects in the non-PVT group. CT/MRI combined with ultrasound were used to diagnose PVT: CT or MRI was used as a confirmative technique when thrombus was suspected by ultrasound.. The exclusion criteria are as follows: (1) chronic liver diseases that did not progress to cirrhosis, such as liver fibrosis; (2) patients with liver failure, hemorrhagic shock, septic shock, and other serious complications; (3) patients with hematological diseases (such as leukemia, multiple myeloma, lymphoma, idiopathic thrombocytopenic purpura); (4) patients with Bud_–_Chiari syndrome; and (5) patients with hepatocellular carcinoma and any other types of cancers, whether or not they had cancer thrombus formation; (6) patients with primary thrombosis in the portal vein without cirrhosis, such as hepatic sinus obstruction syndrome, and thrombophilia; (7) patients with previous thrombosis events, such as pulmonary embolism and deep vein thrombosis; (8) patients taking anticoagulation or thrombolytic drugs within the previous three months. The study protocol was reviewed by the Ethics Committee of the First Affiliated Hospital of Soochow University, and ethical approval was obtained (2021 Ethics approval No. 249).

### 2.3. Data Collection

We retrospectively collected the enrolled patients’ clinical data, including gender, age, etiology of liver diseases, liver disease course, and Child-Pugh score; routine blood tests, such as white blood cell (WBC) count, hemoglobin (Hb) levels, PLT count, mean platelet volume (MPV), and platelet distribution width (PDW); routine biochemical tests on blood, such as alanine aminotransferase (ALT), Alb, and triglycerides (TG); routine blood coagulation tests, such as PT, D-dimer, and antithrombin Ⅲ activity (AT-Ⅲ a); routine imaging data, such as the main portal vein diameter (PVD), absence of spleen, length and thickness of the spleen, esophageal and gastric varices, and ascites; and endoscopy data for esophageal and gastric varices. All clinical data were obtained when PVT was first detected or when the patient was first hospitalized for cirrhosis.

### 2.4. Follow-Up

The time to death was chosen as the main endpoint of the follow-up. In addition, we also counted the incidence of recurrent EGVB, refractory ascites (RA), HE, and liver failure during the follow-up. According to continuous enrolled patients, all patients were followed up by clinical inpatient data, outpatient information, and telephone contact from July to August 2022. The follow-up time was defined as the time from the first hospitalization or first detection of PVT to the time at which the outcome occurred. Recurrent EGVB means that hematemesis or melena occurred after hospitalization with transjugular intrahepatic portosystemic shunt, endoscopic ligation, or conservative treatment to reduce portal pressure. RA is defined as ascites that cannot be eliminated or its early recurrence after paracentesis or that cannot be satisfactorily prevented with sodium restriction and diuretic therapy. [10] The definitions of HE and liver failure were based on the clinical guidelines [11].

### 2.5. Statistical Analysis

SPSS (version 25.0, IBM, Armonk, NY, USA), R software (V.4.2.0, R Foundation for Statistical Computing, Vienna, Austria), and GraphPad Prism 9 (V.9.0.2, GraphPad Software, San Diego, CA, USA) were used for statistical analysis. If the measurement data conformed to a normal distribution, they are expressed as the mean ± standard deviation. If they did not conform to a normal distribution, they are expressed as median and quartile ranges. For the comparison of measurement data between the two groups, the t-test or Mann-Whitney U test was used according to whether the data conformed to a normal distribution. Count data are expressed as frequencies and percentages, and the Pearson χ2 test or Fisher’s exact probability test was used for comparisons between the two groups. R software was used to construct a nomogram to predict the occurrence of PVT in cirrhosis. The performance of the nomogram was assessed by discrimination and calibration curves. The discriminative ability of the model was determined by the area under the receiver operating characteristic curve (ROC), which ranged from 0.5 to 1 (no discrimination to perfect discrimination). The calibration of the prediction model was carried out by a visual curve comparing the predicted and actual probability of PVT. GraphPad Prism 9 was used to construct the survival curve. Unless otherwise stated, all tests were performed at a two-tailed significance level of 0.05. *p* < 0. 05 was considered statistically significant.

## 3. Results

### 3.1. Patient Characteristics

The clinical characteristics of the three cohorts were listed in Table 1. There were no significant differences in gender, age, or etiology between the two groups in the training cohort, while there were significant differences in disease course, Child-Pugh score, Hb, Alb, PT, D-dimer, PVD, spleen length, spleen thickness, splenectomy, esophageal and gastric varices, and ascites between the two groups (*p* < 0.05). Through univariate regression analysis, we chose some significant indicators (*p* < 0. 05) to input into the multivariate regression analysis and found that Alb, D-dimer, splenectomy, esophageal and gastric varices, and PVD were independent risk factors for PVT in cirrhosis (Table 2). In addition, we analyzed the position of PVT among the three cohorts (Table 3). The most common positions of PVT were the main trunk and the left and right branches of the portal vein. There was no significant difference among the groups, with a *p* value of 0.630.

### 3.2. Nomogram Construction

**Figure 2:**
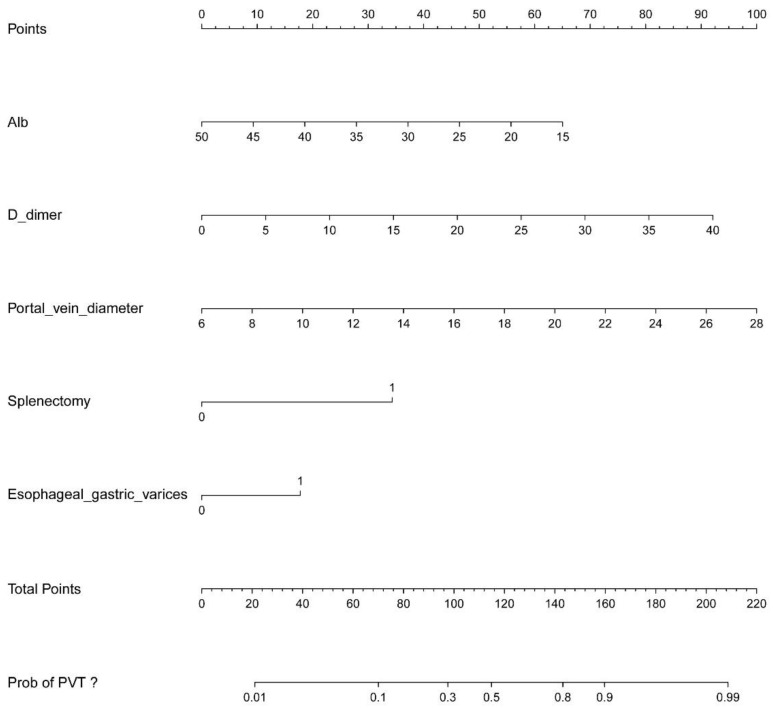
Nomogram of predicting PVT formation based on the independent risk factors. Each variable value has a score on the upper scale axis. The total score can be calculated by summing up each score, then projecting the total score onto the lower total scale axis, and finding the risk probability corresponding to the total score.

**Figure 3:**
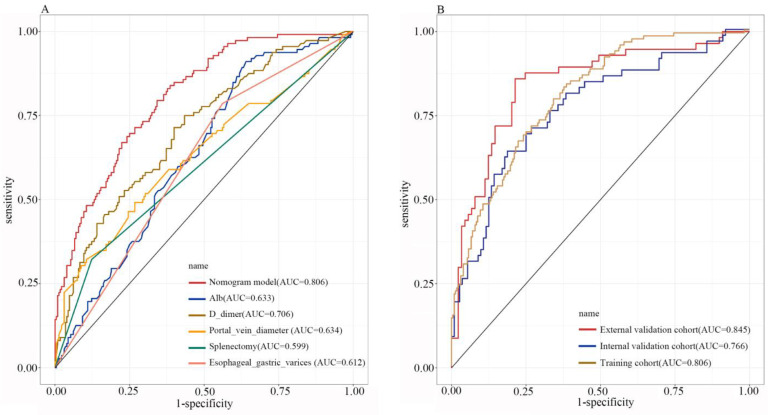
The ROC curves of independent risk factors based on multivariate analysis and nomogram model in the training cohort (**A**). The ROC curves of nomogram used in the three different cohorts (**B**).

**Figure 4:**
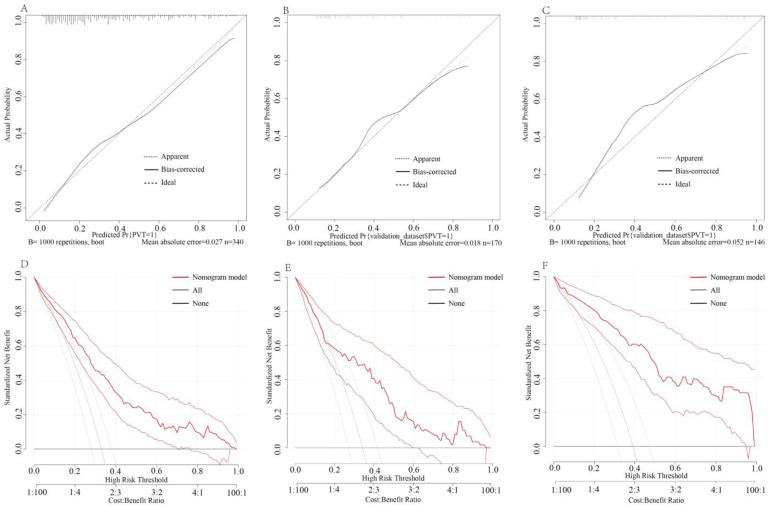
Calibration curves in the training cohort (**A**), internal validation cohort (**B**), and external validation cohort (**C**). The *X*-axis represents the predicted probability, and the *Y*-axis represents the actual probability of PVT. The black solid line indicates the bias correction with bootstrapping (1000 repetitions), and the dotted line represents the training, internal validation, and external validation cohort. The perfect prediction corresponds to the 45° dashed line. The decision curve analysis and 95% CI in training cohort (**D**), internal validation cohort (**E**), and external validation cohort (**F**). The horizontal black line represents the treatment of no patients, the slash gray solid line represents the treatment of all patients, and the red solid line represents the net benefit of the prediction model.

Based on the results of the multivariate analysis, we constructed a nomogram to predict the risk of PVT formation (Figure 2). The nomogram exhibited powerful diagnostic ability in the three cohorts. We used ROC curves to assess the different independent risk factors which were used to construct the predictive model (Figure 3A). With the cutoff value 0.240, the nomogram yielded an accuracy of 0.806 (95% CI, 0.759–0.853; sensitivity, 0.839; specificity, 0.614) in the training cohort. When the cutoff value was 0.404, the nomogram yielded an accuracy of 0.766 (95% CI, 0.688–0.844; sensitivity, 0.638; specificity, 0.812) in the internal validation cohort, and when the cutoff value was 0.240, the nomogram yielded an accuracy of 0.845 (95% CI 0.777–0.913; sensitivity 0.860, specificity 0.787) in the external validation cohort (Figure 3B). The calibration curves revealed good predictive accuracy between the actual probability and predicted probability among the three cohorts (Figure 4A–C). In decision curve analyses, we found that the prediction model had a good clinical benefit compared to the default strategies of treating none or all patients in the three cohorts (Figure 4D–F). In the training cohort, the net benefit of the prediction model showed a superior risk threshold probability compared with the baseline, ranging from 2% to 98%, and in internal and external validation cohorts, both of the net benefits ranged from 4–96%.

### 3.3. Outcome of Follow-Up

**Figure 5:**
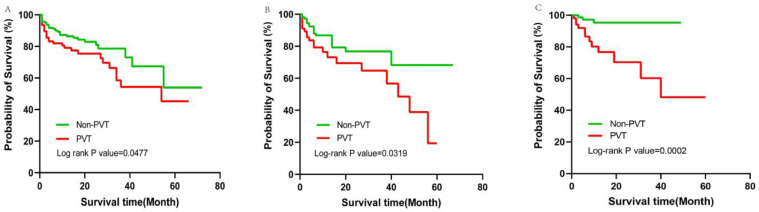
Kaplan–Meier curves in training cohort (**A**), internal validation cohort (**B**), and external validation cohort (**C**). The red line represents the PVT group, and the green line represents the non-PVT group. The *X*-axis indicates the survival time, and the *Y*-axis indicates the probability of survival.

The enrolled patients were followed up from July to August 2022. The Kaplan-Meier survival curves are shown in Figure 5. The median follow-up times of the training, internal validation, and external validation cohorts were 12, 11, and 12 months, respectively. The survival time in the PVT group was shorter than that in the non-PVT group, with log-rank *p* values of 0.0477, 0.0319, and 0.0002 in the training, internal validation, and external validation cohorts, respectively; meanwhile, the median survival times of the PVT group were 54, 43, and 40 months, respectively. We also calculated the incidence of severe complications in all patients during the follow-up, and there was a significant difference between the PVT group and the non-PVT group in recurrent EGVB, with *p* values of 0.009, 0.048, and 0.001 in the three cohorts, respectively. In addition, RA and HE also showed significant differences in the external validation group between the two groups (Table 4).

## 4. Discussion

PVT is one of the common complications of liver cirrhosis. It is commonly believed that patients with liver diseases, especially with advanced cirrhosis, have a tendency to bleed due to deficiency of coagulation factors and hypersplenism. In contrast to previous results, cirrhotic patients are actually more likely to have thromboembolic events due to hypercoagulability with a prothrombotic state and systemic inflammation [3,12]. In our study, we retrospectively analyzed the clinical data from 656 patients and constructed a model to predict PVT formation. The model incorporated laboratory data, imaging features, and endoscopy performance and successfully distinguished high-risk cirrhotic PVT patients from normal cirrhosis patients. Furthermore, the nomogram based on the model had better predictive accuracy than did any of the individual risk factors alone, and it is easy to use in the clinic.

As mentioned above, there are many risk factors for PVT formation in cirrhotic patients. In the present study, we selected five independent risk factors, including Alb, D-dimer, splenectomy, esophageal and gastric varices, and PVD, to build our model. The level of serum albumin can reflect liver synthesis function. A low serum albumin can indicate poor prognosis in patients with liver diseases [13]. Previous studies have shown that serum albumin was negatively correlated with vein thrombosis [14], and decreased albumin levels are associated with an increased risk of venous thromboembolism (VTE) in a linear dose-response manner; this association is independent of the inflammation influence [15]. We found that serum albumin was significantly lower in the PVT group among the three cohorts, and the difference was statistically significant (*p* < 0.001, 0.010, 0.020). Multivariate regression analysis showed that it could be a powerful independent risk factor (*p* = 0.013). Our results are consistent with previous studies which showed that serum albumin was independently associated with PVT after adjustment for several factors, such as Child-Pugh score [6,8,16,17]. However, the underlying mechanism is still unknown. A previous study showed that lower serum albumin would decrease the bioavailability of prostacyclin, which is a potent inhibitor of platelet aggregation [18]. Due to the heparin-like activity of albumin, the anticoagulant effect of antithrombin may be also impaired with the decreased level [19]. Basili’s study showed that low serum albumin was associated with PVT in patients with liver cirrhosis and found that albumin could inhibit agonist-induced platelet activation, which plays an important role in thrombosis formation. Thus, decreased serum albumin may perform as a regulator of the hemostatic system by inhibiting Nox2-mediated oxidative stress and interfering with the platelet activation mechanism [16].

D-dimer is produced during fibrinolysis. It has been widely used for the diagnosis of suspected VTE in clinical practice. A negative D-dimer in combination with clinical manifestations can rule out the diagnosis of deep vein thrombosis and pulmonary embolism, which reduces the need for ultrasound or CT scanning [20,21]. However, there are few articles clarifying the application of D-dimer in cirrhotic patients with PVT. Dai et al. analyzed 21 studies and found that cirrhotic patients with PVT had a significantly higher D-dimer than those without PVT [22], suggesting that D-dimer might be a diagnostic marker for PVT in cirrhosis. Similarly, a large multicenter retrospective cohort analysis showed that PVT had a significantly higher level of D-dimer (2.07 vs. 1.25; *p* < 0.001) in patients with acute worsening of chronic liver disease [23]. In our study, we found that D-dimer was higher in the PVT group than in the non-PVT group, with a significant difference among the three cohorts, which could be an independent risk factor for diagnosis. However, a D-dimer test alone is not specific enough to predict PVT. After analyzing thrombosis in different sites, such as upper extremity thrombosis, cerebral vein thrombosis, and splanchnic vein thrombosis, Ordieres et al. also suggested that D-dimer could not be recommended as a first-line diagnostic tool for thrombosis in unusual sites due to the poorly documented articles [24]. In addition, many pathophysiological processes may lead to increased D-dimer levels. For example, infection will also induce fibrinolysis, and we can find a higher D-dimer level in systemic or local infections, which is regarded as an indicator of a poor prognosis [25]. Patients with tumors, pregnancy, surgery, and other stress reactions usually have hypercoagulable states and elevated D-dimer levels. Thus, we should combine D-dimer with other clinical tools to increase the diagnostic value of PVT.

PVD is considered the best indicator for portal hypertension. The wider the portal vein is, the more likely it is to cause damage to the venous vessel wall due to the extended high pressure in the lumen, and when combined with slow blood flow, the blood easily forms a vortex, promoting coagulation and eventually promoting the formation of PVT [26,27]. Dong et al. found that PVD was the strongest independent risk factor for predicting PVT development (OR: 3.96, AUC: 0.88; *p* < 0.01), with a cutoff of >12.5 mm [7]. Increased PVD is associated with an increased risk of PVT development. From examining patients from two medical centers, Yuan et al. found that PVD and splenic diameter could predict PVT after splenectomy [27]. This is consistent with the results of the present study. PVT is also a complication of intra-abdominal surgery, especially splenectomy. Postsurgical PVT is currently limited to a few small descriptive case series, and a recent study conducted by the Mayo Clinic reported an occurrence rate of PVT after splenectomy was for approximately 8.1% after reviewing datasets from 1745 patients [28]. The mechanism of surgery-associated PVT may be thrombosis provoked by intraoperative vascular manipulation or local postoperative inflammation [29]. In the study of Zhang et al., splenectomy was an independent risk factor for PVT in patients with cirrhosis and acute decompensation [23]. In a retrospective analysis of 113 liver cirrhosis patients without malignancy, splenectomy was identified associated with a 10-fold increased risk of PVT, which was independent to the severity of liver dysfunction [30]. In addition, in our study, we also found that esophageal and gastric varices was independent risk factor for PVT. A prospective trial to identify the impact of PVT in patients with cirrhosis showed that the development of PVT was independently associated with baseline esophageal varices (*p* = 0.01) [8], which is in accordance with our research. Esophageal and gastric varices is considered to be another sign of portal hypertension. A slowed blood velocity could result from increased pressure in the portal vein induced by varicosity. PVT would be more feasible when combined with damage to the endothelium of blood vessels.

## 5. Prognosis and Complications

Cirrhotic patients with PVT tend to have severe complications and poor prognosis. It is generally assumed that PVT contributes to portal hypertension complications, subsequently increasing the risk of mortality. Patients with PVT have more portal hypertension signs, such as EGVB, splenomegaly, and thrombocytopenia. In the present study, we compared the incidence of complications in two groups among three cohorts during the follow-up and found that the occurrence of recurrent EGVB both in the training and validation cohorts showed significant differences (Table 4). In the external validation cohort, we also found that patients with PVT were more prone to HE and RA, suggesting that cirrhotic patients with PVT had severe portal hypertension. From the survival curves, we found that the survival time of the PVT group was shorter than that of the non-PVT group. We assume that patients with PVT have high short-term mortality, which may be caused by severe gastrointestinal bleeding with extremely elevated portal vein pressure, septic shock due to immunosuppression, dysregulation of the intestinal microbiota, and intestinal ischemic necrosis due to complete obstruction of the portal vein and mesenteric vein, eventually leading to multiple organ failure. The same was found in the study of Cool et al., who reported that PVT was associated with increased mortality, implying that PVT has a significant impact on the cirrhotic prognosis [31]. However, recent findings suggested that the development of PVT was an indicator but not a direct cause of the progression of liver disease [8,32]. PVT was not independently associated with the progression of liver disease or death, and thus other factors may also be involved. It is difficult to determine whether the presence of PVT causes greater portal hypertension and an exacerbation of cirrhosis, or whether with the progression of liver cirrhosis, the portal vein pressure increases and leads to a higher probability of PVT, eventually causing death in PVT patients. The contribution of PVT to hepatic decompensation and overall mortality in cirrhosis is less clear.

There are some limitations in our study. First, this study retrospectively collected the patients’ data; thus, the reliability and completeness of the data are limited. In addition, the enrolled patients came from one region, and the etiology of cirrhosis and lifestyle factors, such as diet and alcohol consumption, may also affect the development of PVT. Third, considering the changes in the clotting system, inflammatory response, and platelet function in PVT, coagulation factors, fibrinolysis markers, inflammation biomarkers, and activation markers of platelets could also be used to predict PVT. Finally, the sample size of our research is small, and we only included patients from two medical centers. Research with a large sample size and patients from additional medical centers should be carried out in the near future.

## 6. Conclusions

The model we constructed to predict PVT in cirrhotic patients has good diagnostic value. Patients with PVT have poor prognosis due to portal hypertension. With this prediction model, clinicians can evaluate patients with cirrhosis for their risk of developing PVT and apply the appropriate interventions as early as possible. For example, for patients with high scores on the nomogram, the lumen and blood flow of the portal vein should be assessed periodically by ultrasound. Symptomatic supportive treatment such as albumin infusion, the reduction of portal hypertension, or even prophylactic anticoagulation could be given after evaluation of the hemorrhage risk and systemic status. The proper management of PVT requires early detection, diagnosis, treatment, and follow-up. We expect that this nomogram based on laboratory tests and imaging findings will be a useful tool to guide individual care for high-risk cirrhotic patients.

## Figures and Tables

**Table 1:** Basic characteristic in training and validation cohorts.

	Training Cohort				Internal Validation Cohort				External Validation Cohort			
	Total (n = 340)	Non-PVT (n = 228)	PVT (n = 112)	*p* Value	Total (n = 170)	Non-PVT (n = 112)	PVT (n = 58)	*p* Value	Total (n = 146)	Non-PVT (n = 89)	PVT (n = 57)	*p* Value
Male	193	127	66	0.572	105	70	35	0.784	99	61	38	0.813
Age(year)	59.06 ± 13.42	58.86 ± 14.17	59.49 ± 11.78	0.663	60.74 ± 13.12	60.96 ± 13.86	60.31 ± 11.65	0.759	56.90 ± 12.16	55.38 ± 12.19	59.26 ± 11.83	0.060
Disease course(year)	6.00 (1.00, 10.00)	5.00 (1.00, 10.00)	8.00 (3.00, 10.00)	0.005	8.50 (1.00, 10.00)	6.00 (1.00, 10.00)	10.00 (4.75, 10.00)	0.004	2.00 (1.00, 5.25)	1.00 (1.00, 4.00)	4.00 (1.00, 8.00)	0.002
Etiology												
Viral hepatitis	149	104	45	0.342	64	40	24	0.470	97	58	39	0.685
Schistosoma hepatitis	48	27	21	0.086	32	24	8	0.227	-	-	-	-
Autoimmune hepatitis	43	28	15	0.772	25	17	8	0.809	15	9	6	0.936
Alcohol hepatitis	25	19	6	0.323	15	10	5	0.947	18	11	7	0.989
Others ^1^	75	50	25	0.935	34	21	13	0.571	16	11	5	0.498
Child–Pugh Score	8.00 (6.00, 9.00)	8.00 (6.00, 9.00)	8.00 (7.00, 10.00)	0.008	8.00 (7.00, 9.00)	8.00 (6.00, 9.00)	8.00 (7.00, 9.25)	0.672	8.00 (6.00, 9.00)	7.00 (5.00, 8.00)	8.00 (7.00, 10.00)	0.001
WBC (×10^9^/L)	5.8 ± 3.9	5.63 ± 3.77	6.15 ± 4.15	0.248	5.75 ± 3.75	5.37 ± 3.02	6.50 ± 4.80	0.106	4.34 ± 2.52	3.94 ± 1.58	4.95 ± 3.44	0.042
PLT (×10^9^/L)	78.50 (50.00, 129.25)	78.50 (51.00, 122.75)	78.00 (45.25, 146.75)	0.814	77.5 (50.00, 117.00)	75.50 (50.00, 103.50)	86.50 (47.25, 161.25)	0.166	77.50 (51.00, 152.75)	75.00 (48.50, 124.50)	86.00 (54.50, 191.00)	0.124
PDW (%)	15.83 ± 2.46	15.86 ± 14.17	15.77 ± 2.12	0.771	15.61 ± 2.14	15.85 ± 2.05	15.17 ± 2.24	0.057	14.53 ± 3.03	14.69 ± 2.76	14.29 ± 3.42	0.495
MPV (fL)	11.35 ± 1.69	11.35 ± 1.68	11.34 ± 1.73	0.977	11.17 ± 1.52	11.21 ± 1.56	11.09 ± 2.24	0.639	11.26 ± 1.34	11.35 ± 1.30	11.12 ± 1.41	0.372
Hb (g/L)	97.24 ± 29.07	99.78 ± 29.81	92.08 ± 26.89	0.021	100.57 ± 29.95	102.37 ± 30.71	97.10 ± 28.36	0.278	112.10 ± 33.07	119.63 ± 33.60	100.33 ± 28.74	<0.001
ALT (U/L)	24.10 (16.33, 38.28)	25.00 (17.25, 39.18)	22.75 (14.45, 37.15)	0.177	22.85 (15.55, 33.80)	22.75 (15.78, 39.00)	23.55 (15.38, 30.65)	0.643	30.50 (21.00, 61.00)	38.00 (24.00, 99.00)	25.00 (18.50, 37.50)	<0.001
Alb (g/L)	31.18 ± 5.62	32.07 ± 5.87	29.38 ± 4.58	<0.001	30.64 ± 5.86	31.36 ± 6.52	29.24 ± 4.00	0.010	33.20 ± 6.22	33.56 ± 6.98	31.42 ± 3.93	0.020
TG (μmol/L)	1.04 ± 0.66	1.06 ± 0.69	0.99 ± 0.60	0.312	1.08 ± 0.69	1.13 ± 0.70	0.97 ± 0.78	0.147	1.10 ± 0.73	1.28 ± 0.83	0.78 ± 0.32	<0.001
PT (s)	15.96 ± 3.19	15.64 ± 2.89	16.62 ± 3.66	0.007	15.84 ± 4.13	16.01 ± 4.71	15.51 ± 2.70	0.459	13.96 ± 2.64	13.61 ± 2.72	14.50 ± 2.42	0.046
AT-IIIa (%)	59.15 ± 19.41	60.14 ± 2.89	57.13 ± 18.23	0.178	59.67 ± 19.59	61.38 ± 20.27	56.36 ± 17.89	0.113	65.04 ± 21.95	66.81 ± 21.69	62.26 ± 22.26	0.222
D-dimer (μg/mL)	1.88 (0.72, 4.09)	1.20 (0.55, 3.03)	3.40 (1.47, 8.60)	<0.001	1.99 (0.69, 4.23)	1.33 (0.54, 3.11)	3.25 (1.71, 6.67)	<0.001	1.35 (0.39, 3.39)	0.58 (0.21, 2.27)	3.16 (1.62, 6.46)	<0.001
PVD (mm)	13.56 ± 3.09	13.02 ± 2.56	14.67 ± 3.74	<0.001	13.91 ± 3.37	13.08 ± 2.89	15.51 ± 3.65	<0.001	14.03 ± 2.84	13.27 ± 2.09	15.22 ± 3.42	<0.001
Ascites	189	112	77	0.001	104	64	40	0.134	78	30	48	<0.001
Esophageal gastric varices	216	128	88	<0.001	107	62	45	0.004	84	32	52	<0.001
Splenectomy	64	28	36	<0.001	26	12	14	0.021	21	1	20	<0.001
Spleen thickness(mm)	51.75 ± 14.12	50.06 ± 13.17	56.18 ± 15.58	0.001	51.03 ± 11.44	49.78 ± 11.44	53.89 ± 11.05	0.047	43.69 ± 12.78	41.70 ± 12.60	47.49 ± 12.42	0.025
Spleen length(mm)	140.88 ± 36.12	136.15 ± 35.32	153.32 ± 35.45	<0.001	141.94 ± 33.32	140.32 ± 30.68	145.61 ± 38.78	0.382	140.71 ± 34.90	137.65 ± 33.62	146.59 ± 37.00	0.208

^1^ Others: steatohepatitis cirrhosis, cholestatic liver disease, combined two or more etiology cirrhosis, and unexplained cirrhosis.

**Table 2:** Univariate and multivariate regression analysis in the training cohort.

	Univariate Regression Analysis				Multivariate Regression Analysis			
	Wald Value	*p* Value	OR	95% CI	Wald Value	*p* Value	OR	95% CI
Child–Pugh Score	7.342	0.007	1.163	1.043, 1.298	0.290	0.590	0.939	0.747, 1.180
PT	6.778	0.009	1.009	1.024, 1.180	0.831	0.362	1.047	0.948, 1.157
Hb	5.210	0.022	0.991	0.983, 0.999	0.283	0.594	1.003	0.992, 1.014
Alb	16.286	<0.001	0.908	0.867, 0.952	6.158	0.013	0.911	0.847, 0.981
D-dimer	23.328	<0.001	1.136	1.079, 1.197	13.607	<0.001	1.116	1.053, 1.184
PVD	19.205	<0.001	1.195	1.103, 1.294	20.350	<0.001	1.241	1.130, 1.363
Splenectomy	18.196	<0.001	3.383	1.933, 5.924	23.658	<0.001	5.512	2.771, 10.966
Spleen length	11.823	0.001	1.014	1.006, 1.021	0.909	0.340	1.006	0.994, 1.018
Spleen thickness	9.779	0.002	1.030	1.011, 1.050	0.055	0.815	1.004	0.974, 1.034
Ascites	11.475	0.001	2.279	1.415, 3.670	0.580	0.446	1.300	0.662, 2.553
Esophageal gastric varices	15.635	<0.001	2.865	1.700, 4.826	6.708	0.010	2.424	1.240, 4.738

**Table 3:** The comparison of position of thrombosis in the different cohorts.

	Training Cohort	Internal Validation Cohort	External Validation Cohort	χ^2^	*p* Value
Total	112	58	57	6.188	0.630
Main trunk and branches of portal vein	74 (66.07%)	37 (63.79%)	31 (54.39%)		
Portal vein and splenic vein	5 (4.46%)	6 (10.34%)	4 (7.02%)		
Portal vein and superior mesenteric vein	22 (19.64%)	11 (18.97%)	13 (22.80%)		
Splenic vein or superior mesenteric vein	3 (2.68%)	1 (1.72%)	4 (7.02%)		
All ^1^	8 (7.12%)	3 (5.17%)	5 (8.77%)		

^1^ All: The whole portal vein system including main trunk and branches of portal vein, along with the splenic vein and superior mesenteric vein being filled with thrombosis.

**Table 4:** Incidence of severe complications during the follow-up.

	Training Cohort				Internal Validation Cohort				External Validation Cohort			
	Total	Non-PVT	PVT	*p* Value	Total	Non-PVT	PVT	*p* Value	Total	Non-PVT	PVT	*p* Value
Recurrent EGVB (n=)	67	35	32	0.009	38	19	19	0.048	29	10	19	0.001
RA (n=)	59	35	24	0.172	28	18	10	0.850	52	25	27	0.016
HE (n=)	25	17	8	0.528	18	9	9	0.269	22	8	14	0.009
Liver failure (n=)	9	5	4	0.286	6	5	1	0.590	13	6	7	0.259

## Data Availability

All data are within the manuscript.
